# A Study on the Impact of Brand Ritual on Online Word-of-Mouth Communication

**DOI:** 10.3390/bs15030278

**Published:** 2025-02-26

**Authors:** Tao Wen, Ziwei Wang, Shuang Wang

**Affiliations:** School of Economics and Management (School of Tourism), Dalian University, Dalian 116622, China; wentao@dlu.edu.cn (T.W.); wziwei1998@sina.com (Z.W.)

**Keywords:** brand ritual, online word-of-mouth communication, flow experience, consumer–brand relationship norms

## Abstract

The study aims to explore the impact mechanism of brand ritual on online word-of-mouth communication, introducing the mediating variable—flow experience—and the moderating variable—consumer–brand relationship norms. The study uses the approach of the experimental research. In Experiment 1, with the watch as the experimental product and the advertisement as the online scene, 62 subjects in the pre-experiment and 132 subjects in the formal experiment are recruited to verify the main effect of brand ritual on online word-of-mouth communication. In Experiment 2, with the tea bag as the experimental product and the online press conference as the online scene, 73 subjects in the pre-experiment and 185 subjects in the formal experiment are recruited to verify the mediating role of flow experience in the impact of brand ritual on online word-of-mouth communication. In Experiment 3, with the scented candle as the experimental product and the promotional video of the e-commerce store as the online scene, 81 subjects in the pre-experiment and 269 subjects in the formal experiment are recruited to verify the moderating role of consumer–brand relationship norms in the impact of brand ritual on online word-of-mouth communication/flow experience. The results show that brand ritual is more effective in promoting online word-of-mouth communication than random action, flow experience plays a completely mediating role in the impact of brand ritual on online word-of-mouth communication, and consumer–brand relationship norms play a moderating role in the impact of brand ritual on online word-of-mouth communication/flow experience. The study not only reveals the impact mechanism of brand ritual on online word-of-mouth communication, but also provides strong guidance for companies to utilize brand ritual, flow experience, and consumer–brand relationship norms to promote online word-of-mouth communication.

## 1. Introduction

The 53rd Statistical Report on Internet Development in China showed that by December 2023, the number of Internet users in China had reached 1.092 billion, and the Internet penetration rate had reached 77.5% (China Internet Network Information Center, 2024). While the Internet affects people’s lives, it also gives birth to online word-of-mouth communication. More and more consumers are sharing their feelings about products and services through online platforms. From the popularity of Soy Sauce Latte, whose daily sales exceeded 5.42 million cups, to the popularity of South Potato in Harbin, the Internet spreads products, services, and other information at a rapid pace, creating a huge network effect. However, in the era of information overload, how to effectively promote online word-of-mouth communication has become a common concern in the marketing field. Fortunately, brand ritual, as a symbolic activity that contains brand meaning, is becoming a new tool to promote online word-of-mouth communication. For example, when consumers watch the Oreo cookies’ unique eating-method advertisement “twist, lick, dunk”, brand ritual conveys the concept of “fun and healthy”, thus constituting a memorable experience and triggering an online word-of-mouth effect. Therefore, it is necessary for this study to explore the impact mechanism of brand ritual on online word-of-mouth communication.

The following study consists of literature reviews and research hypotheses, experimental design and data analysis, discussion, and conclusions. In the first part, the study puts forward research hypotheses and constructs a theoretical model on the basis of related research of brand ritual, online word-of-mouth communication, flow experience, and consumer–brand relationship norms. In the second part, the study designs three experiments to verify these research hypotheses. Experiment 1 takes the watch as the experimental product and the advertisement as the online scene, recruiting subjects in the pre-experiment and formal experiment to verify the main effect of brand ritual on online word-of-mouth communication. Experiment 2 takes the tea bag as the experimental product and the online press conference as the online scene, recruiting subjects in the pre-experiment and formal experiment to verify the mediating role of flow experience in the impact of brand ritual on online word-of-mouth communication, as well as the result of Experiment 1. Experiment 3 takes the scented candle as the experimental product and the promotional video of the e-commerce store as the online scene, recruiting subjects in the pre-experiment and formal experiment to verify the moderating role of consumer–brand relationship norms in the impact of brand ritual on online word-of-mouth communication/flow experience, as well as the results of Experient 1 and 2. In the third part, the study summarizes and discusses research findings, pointing out managerial implications, research limitations and prospects. In the fourth part, the study draws research conclusions.

## 2. Literature Review and Research Hypotheses

### 2.1. Literature Review

#### 2.1.1. Brand Ritual

Ritual originated from the Latin word-ritus and emerged as a research term in the 19th century, gradually applied to the field of consumption at the end of the 20th century. Rook (1985) first proposed the definition of ritual in consumer behavior, which refers to a type of expressive, symbolic activity constructed of multiple behaviors that occur in a fixed, episodic sequence, and that tend to be repeated over time.

With the continuous deepening of ritual theory and the diversification of its application scenarios, brand ritual has emerged. Scholars have given different definitions of the concept of brand ritual from different perspectives (Wen & Wang, 2024).

Prexl and Kenning (2011) defined brand ritual as regularly occurring acts, proceeding in a largely identical and therefore standardized fashion. Fang (2011) proposed that in brand rituals, the brand consciously cooperate with the rituals to create a pleasant and sacred atmosphere, thereby guiding customers to consume. Raj (2012) defined brand ritual as a ritual activity added to the basis of brand consumption habits, which is a series of repetitive, meaningful, and nonfunctional behaviors that occur in the process of brand and customer connection. Based on the research of Raj (2012), Xue (2015) highlighted the study of interactive characteristics of brand ritual and defined it as ritualized interactive behaviors between brands and consumers. Ran and Wei (2017) expanded nonfunctionality to expressing brand value and meaning. Ran and Wan (2023) thought that brand ritual refers to the repetitive, symbolic, and nonutilitarian actions associated with a brand. Based on previous scholars’ research and practical consumption scenarios, brand ritual in this study is defined as a ritualistic activity designed and constructed by the brand to enhance interaction and connection with consumers, and to convey value and meaning.

#### 2.1.2. Online Word-of-Mouth Communication

Word-of-mouth is one of the oldest ways to convey information. Katz and Lazarsfeld (1955) described it as the exchange of information between consumers, which plays an important role in changing consumers’ attitudes towards products and services and shaping their behavior. Arndt (1967) first proposed a clear definition of word-of-mouth from the perspective of marketing, stating that word-of-mouth is any verbal and personal communication about the brand, product, service, or organization, whether positive or negative, in which the information receiver perceives the sender to have non-commercial intentions. Westbrook (1987) defined word-of-mouth as informal communication between consumers regarding the use or characteristics of specific goods and services. Barreto (2014) defined word-of-mouth as the process of informal communication between information senders and individuals or multiple recipients who share or acquire information. Based on the definitions of word-of-mouth by the three scholars above, word-of-mouth can be regarded as an informal communication between the subject (sender and receiver of information) regarding the object (product, service, brand, or organization), used for the non-commercial purposes of sharing and obtaining information.

The emergence of Web 2.0 and new media channels has expanded the concept of word-of-mouth to online environments with a wider audience, giving rise to online word-of-mouth and becoming one of the most influential sources of information on the internet. Hennig-Thurau et al. (2003) defined online word-of-mouth (electronic word-of-mouth) as the positive or negative statements made by potential or actual consumers to products or companies, which are transmitted to others through the Internet. Huang (2016) believed that online word-of-mouth refers to consumers spreading positive or negative information about a product or service through social media. Xiong (2024) believed that online social network word-of-mouth is a type of online word-of-mouth, specifically referring to the informal communication behavior between consumers about products or services through social platforms, which includes consumer evaluations, experiences, and comments. Combined with the definition of online word-of-mouth communication by scholars, it can be defined as the information dissemination between subjects (information sender and receiver) on objects (products, services, etc.) through the Internet (social media, etc.).

#### 2.1.3. Flow Experience

Flow experience was initially proposed by the renowned psychologist Csikszentmihalyi, who described flow in his book “Flow: The Psychology of Optimal Experience” as a state in which a person is completely immersed in a certain activity, ignoring the existence of other things (Csikszentmihalyi, 1990).

As a positive psychology method for understanding optimal experience, flow experience has attracted widespread attention from scholars. Some scholars have adopted Csikszentmihalyi’s definition, viewing flow experience as a psychological state. For example, Decloe et al. (2009) defined flow experience as a state of optimal arousal often spurred by a balance of challenge and skill during an activity. Bilgihan (2016) believed that flow experience is the perceived state of enjoyment, concentration, and control. Lin and Yu (2019) believed that online flow experience is the cognitive state that consumers feel in an online setting. In addition, a series of positive factors triggered by flow experience will further affect consumers’ willingness to consume (Du & Zhu, 2024). In order to more accurately describe flow experience triggered by brand ritual, combined with the characteristics of online consumption scenarios, the study defines flow experience as the psychological state of total devotion, concentration, and positive pleasure generated by consumers when they participate in activities.

#### 2.1.4. Consumer–Brand Relationship Norms

Since the early research of Sherif (1936), the concept of norms has been at the heart of research in the social sciences, including social psychology, political science, law, and economics. Although the literature on this concept is diverse, its basic meaning is common. Heide and John (1992) refined the previous concept and defined it as the expectation of group members for a certain behavior.

In the 1990s, the development of related studies such as relationship marketing led scholars to believe that the relationship between consumers and brands is not only a simple interest exchange relationship, but also a two-way and dynamic social-interaction relationship. For example, Fournier (1998) proposed that the relationship types between consumers and brands are similar to best friends, partners, and so on in interpersonal relationships. Moon (2000) proposed that consumers apply interpersonal norms or social relationships to their attitudes towards enterprises or brands. Aggarwal (2004) also indicates that there are the deep-level relationship norms between consumers and brands, which are consumer–brand relationship norms. Based on the classification of interpersonal relationships by Clark and Mils (1993), Aggarwal (2004) divided consumer–brand relationship norms into exchange relationship norms and communal relationship norms. Under exchange relationship norms, consumers tend to consider brands as service/product providers and to place a higher focus on brand performance and outcomes; under communal relationship norms, consumers feel responsible for their partners’ welfare and are willing to respond positively when a partner needs help (S. Lee & Kim, 2024).

### 2.2. Research Hypotheses

#### 2.2.1. The Main Effect of Brand Ritual on Online Word-of-Mouth Communication

Malefyt (2015) conducted an ethnographic investigation on male shaving rituals, and the results showed that consumers share their self-constructive shaving rituals through the brand community to meet the socialized construction of self-identity. Wei et al. (2020) proposed that when brand rituals are presented as visual materials in front of consumers, they can also activate consumers’ perception of the rituals and increase consumers’ trust in the brand. Whether it is the self-identity needs generated by consumers’ actual participation or the trust perception generated through watching brand rituals, these positive psychological perceptions are important guarantees for consumers to make subsequent behaviors and also important antecedents for consumers to generate online word-of-mouth communication behavior.

Previous studies have shown that companies can make social exchange commitments to consumers through the current behavior and situational clues of brand ritual, thereby increasing consumers’ trust and love for the brand (Wei et al., 2020; Cruz-Ros et al., 2024). As an important factor affecting online word-of-mouth communication, the increase in brand trust and love will also enhance their willingness to spread online word-of-mouth.

In summary, the study proposes the following research hypothesis:

**H1.**  *Compared to random action, brand ritual is more effective in promoting online word-of-mouth communication.*

#### 2.2.2. The Mediating Role of Flow Experience in the Impact of Brand Ritual on Online Word-of-Mouth Communication

Flow experience arises from the dynamic process of interaction between individuals and their environment, and is a positive experience triggered by individuals focusing their limited attention on the current interaction (Csikszentmihalyi & Larson, 2014). Rufi et al. (2016) demonstrated that in the collective rituals, subjects’ cognitions, the action, and the external feedback received while performing the activity reach consensus, and the loss of self-consciousness creates a heightened sense of belonging, leading to the state of flow experience. E. M. Lee et al. (2016) pointed out that in extreme rituals, individuals’ high levels of attention and skills contribute to the generation of flow experience. Similarly, brand ritual also has procedural steps, task clues that can be completed and manipulated, and experiences that trigger positive emotions included in the above rituals, providing complete conditions for the generation of flow experience.

Since Hoffman and Novak (1996) introduced flow experience into online consumption scenes, flow experience has become a state of consumer experience that is different from daily life, providing motivation for consumers’ subsequent behaviors. Scholars have demonstrated the direct relationship between flow experience and online word-of-mouth communication. For example, Renard (2013) found that flow experience depends on the balance between the perceived skill ability and the perceived challenge, and positively affects personal information sharing and word-of-mouth communication with the brand. Jin and Tian (2016) pointed out that flow experience has a direct positive impact on consumer’s WOM intention when exploring the influence of website characteristics on consumer’s WOM intention. Y. Y. Li and Wang (2021) explored the impact of customer perceived value on online word-of-mouth communication, using flow experience as a mediating variable, and pointed out that flow experience generated by consumers during online shopping drives online word-of-mouth communication.

In summary, the study proposes the following research hypotheses:

**H2.**  *Flow experience plays a mediating role in the impact of brand ritual on online word-of-mouth communication.*

**H2a.**  *Compared to random action, brand ritual can better stimulate flow experience.*

**H2b.**  *Flow experience promotes online word-of-mouth communication.*

#### 2.2.3. The Moderating Role of Consumer–Brand Relationship Norms in the Impact of Brand Ritual on Online Word-of-Mouth Communication/Flow Experience

There are the deep-level relationship norms between consumers and brands (consumer–brand relationship norms). Previous studies have shown that consumer–brand relationship norms have a significant positive impact on consumer interaction and relationship building. For example, Simon (2017) proposed that consumers interact with brands under the guidance of relationship norms, selecting brand information during these interactions and assessing the brand’s conduct. Lu (2019) pointed out in his study on the effectiveness of online apology patterns that compared to exchange relationship norms, the impact of apology patterns under communal relationship norms on post-recovery satisfaction is more significant. Fu (2021) found that consumer–brand relationship norms play a moderating role in the impact of social perception evaluation on consumers’ willingness to forgive when studying the impact of the use of online emoticons on consumers’ forgiveness. Therefore, the study believes that communal and exchange relationship norms play a moderating role in the impact of brand ritual on online word-of-mouth communication.

Kapitány and Nielsen (2015) proposed that, causally, the opacity of the ritual means that the actions that make up the ritual do not cause actual physical changes in the real world, but rather refer to the impact of the meaning contained in the ritual on individual psychological cognition. This viewpoint is consistent with the nonfunctional characteristics of the ritual, and also indicates that consumers under different consumer–brand relationship norms may receive different emotional benefits from brand ritual. Specifically, consumers under exchange relationship norms are more concerned about their own benefits and costs, and the emotional resources provided by brand rituals are far less effective than substantial material rewards, which cannot promote consumers to make positive behaviors towards the brand. Therefore, the impact of brand ritual on online word-of-mouth communication is not significant. However, consumers under communal relationship norms may pay more attention to the emotional resources they obtain in interacting with the brand. Symbolic meaning and emotional care provided by brand ritual can promote positive reactions of consumers and online word-of-mouth communication of the brand.

In addition, as a moderated mediator variable, flow experience is moderated by different consumer–brand relationship norms, resulting in varying levels of flow experience intensity and playing a mediating role in the impact of brand ritual on online word-of-mouth communication. An important antecedent of flow experience is personal characteristics (personal perception and individual differences). Therefore, consumers under different relationship norms may perceive varying levels of flow experience intensity from brand ritual. Specifically, consumers under exchange relationship norms are unable to obtain substantial and immediate benefits from brand ritual, making it difficult for them to perceive hedonic value provided by brand ritual. As a result, their flow experience intensity is greatly reduced, and the impact of brand ritual on online word-of-mouth communication is not different from that of random action. In contrast, consumers under communal relationship norms are better able to obtain positive emotions such as pleasure, experience, and immersion from brand ritual, greatly increasing the intensity of flow experience and thus enhancing their willingness to spread online word-of-mouth. C. Y. Li (2023) also found that consumer–brand relationship norms (communal or exchange relationship norms) can moderate the impact of ritual consumption on self-brand connection.

In summary, the study proposes the following research hypotheses:

**H3.**  *Consumer–brand relationship norms play a moderating role in the impact of brand ritual on online word-of-mouth communication/flow experience.*

**H3a.**  *Under communal relationship norms, brand ritual is more effective in promoting online word-of-mouth communication than random action; under exchange relationship norms, there is no significant difference in the impact of random action and brand ritual on online word-of-mouth communication.*

**H3b.**  *Under communal relationship norms, brand ritual is more effective in stimulating flow experience and promoting online word-of-mouth communication than random action; under exchange relationship norms, there is no significant difference in the impact of random action and brand ritual on flow experience.*

**Figure 1:**
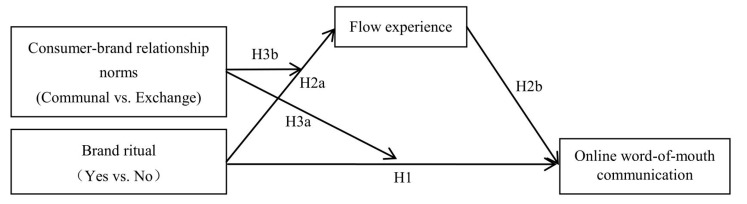
The theoretical model.

Based on the above research hypotheses, the study constructs a theoretical model (Figure 1).

## 3. Experimental Design and Data Analysis

According to the above research hypotheses, the study conducts experimental design, implementation, and data analysis. The experimental products are determined through pretest. The main effect of brand ritual on online word-of-mouth communication, the mediating role of flow experience, and the moderating role of consumer–brand relationship norms are repeatedly verified through three experiments, which include the pre- experiment and the formal experiment, respectively.

### 3.1. Experimental Products

Empirical research on brand ritual is mostly conducted through experimental methods, and the selection of experimental products is also diverse. For example, Wei et al. (2020) used running shoes and pens as experimental products in their study on the impact of brand rituals on consumer trust. Ran and Wan (2023) used fruit tea, fruit juice, a pen, and hand cream as experimental products when studying the interactive effect of brand ritual and brand personality on consumer purchase. To avoid interference with the research results caused by the selection of experimental products, the study determines the product categories for the next three experiments through experimental pre test.

A total of 35 participants (15 males and 20 females) are randomly invited to participate in the experimental pretest. Firstly, 35 participants are asked to answer whether they have experienced a sense of the ritual during the consumption process. After screening, a final sample of 33 participants (13 males and 20 females) is determined. All 33 participants are asked to recall and write down two–four products/brands that have triggered their sense of the ritual more frequently. After the pretest, the products and brands filled in by these participants are classified and preliminarily screened for product categories with a frequency not less than 10 times. The specific situation is shown in Table 1.

Based on the frequency of appearance of each product category in Table 1, tea, scented candle, and watch are preliminarily proposed as the follow-up experimental products. Considering the operability and feasibility of the follow-up experiments, tea is concretized as tea bag, and in order to eliminate the influence of prior experience of the subjects on the research results in the follow-up experiments, brand virtualization is carried out on the selected three experimental products. Finally, three experimental brand products are determined, which are the “Fugit” watch (Experiment 1), the ”Mingting” tea bag (Experiment 2), and the “Xiangyun” scented candle (Experiment 3).

### 3.2. Experiment 1

Experiment 1 adopts a single-factor intergroup design (brand ritual vs. random action) to test the main effect of brand ritual on online word-of-mouth communication.

#### 3.2.1. Pre-Experiment in Experiment 1

The pre-experiment aims to test the effectiveness of brand ritual manipulation when using the watch as the experimental product and the advertisement as the online scene. This manipulation method draws on the research of Ran (2017) and Wei et al. (2020). The experimental group (brand ritual group) and the control group (random action group) use the same brand introduction. The materials are as follows:

**Fugit** is a French watch brand founded in 1991. Since its establishment, it has always adhered to the brand philosophy of “time is the most precious gift”, and its product design integrates unique creativity and superb craftsmanship. Recently, the brand has launched a series of watches called Voice of Time, aiming to convey the concept of “cherishing time and grasping the present” to consumers, and has created a new product advertisement.

Subsequently, there is manipulation of the ritual, and in order to avoid the influence of the workload of the subjects on the experimental results, the word count of both sets of materials is strictly controlled at 74 words. Vohs et al. (2013) found that ritualistic behavior can enhance the enjoyment of consumption experience compared to random action. Therefore, the brand ritual group enhances subjects’ ritual perception through the expression of ritualistic behavior, while the random action group improves subjects’ sense of randomness through expressions such as “recommend” and “should”. The materials are as follows:

**Brand Ritual Group:**

Calm down, gently place the watch on your palm.

Close your eyes and feel the temperature of the watch strap, and use your fingers to feel the arc and texture of the strap.

Open your eyes, gaze at the second hand turning and listen attentively to its sound.

Follow the ticking sound of the second hand, count from one to ten.

Wear the watch; rotate your wrist to adjust it to the optimal position.

**Random Action Group:**

It is recommended to wear the watch on your non-dominant hand to reduce friction.

It is recommended to wear the watch one to two centimeters behind the wrist bone, lifting the wrist without pushing against it.

When wearing the watch, observe whether the second hand, minute hand, and hour hand move normally.

You should avoid placing the watch next to electrical appliances and objects generating magnetic fields.

Clean the watch strap and case regularly.

The pre-experiment recruited 62 subjects (27 males and 35 females) through a paid recruitment platform (Credamo), who all passed attention detection. The subjects were randomly divided into two groups: 29 in the brand ritual group and 33 in the random action group. The subjects first read the brand introduction, and then the online scene of watching the product advertisement was created for them. Then, they were presented with experimental materials of brand ritual (vs. random action). Afterwards, the subjects answered relevant questions, including attention detection questions, ritual perception questions, and demographic information questions. Ritual perception questions refer to the research of Ran (2017), and the scale uses the 7-point Likert scale (1 = strongly disagree, 7 = strongly agree). The analysis results of independent-samples t-test show that the mean value of ritual perception in the brand ritual group was significantly higher than that in the random action group (M_brand ritual group_ = 5.391, M_random action group_ = 4.081, t = 4.218, *p* < 0.05), and the mean value of random perception in the brand ritual group was significantly lower than that in the random action group (M_brand ritual group_ = 2.55, M_random action group_ = 3.42, t = −2.041, *p* < 0.05). The manipulation of brand ritual experimental materials was initially successful.

#### 3.2.2. Formal Experiment in Experiment 1

Xie (2021) believed that negative emotions such as anxiety, sadness, and being controlled can have an impact on the experiment. To avoid the potential impact of negative emotions on the subjects, negative emotions are measured before the start of the experiment. Subsequently, the subjects are randomly divided into two groups (brand ritual vs. random action) and read relevant experimental materials separately. Finally, the subjects fill out the questionnaire items based on the materials, including attention detection, ritual perception, online word-of-mouth communication, and demographic information. Each item is measured using the 7-point Likert scale.

The measurement of negative emotions is designed based on the research of Xie (2021) and Watson et al. (1988), with a total of 5 items (Table 2).

The measurement of ritual perception is designed based on the research of Ran (2017), with a total of 3 items (Table 3). 

The measurement of online word-of-mouth communication is designed based on the research of Huang (2016) and Wang (2021), with a total of 5 items (Table 4).

A total of 132 subjects are recruited through a paid recruitment platform (Credamo); the platform’s function of filtering specific questionnaire users is used to ensure that the subjects in the pre-experiment cannot participate in this experiment. Excluding questionnaires that fail attention detection, 131 valid questionnaires are obtained (66 in the brand ritual group and 65 in the random action group). Firstly, the brand ritual group and random action group are coded (0 = random action group, 1 = brand ritual group). Secondly, the negative emotion measurement items, ritual perception measurement items, and online word-of-mouth communication measurement items are averaged. Finally, data analysis is conducted using SPSS 26.0.

- Descriptive statistical analysis

Descriptive statistical analysis is conducted on gender, age, education, occupation, and monthly average consumption of the subjects. From the perspective of subject gender, the male-to-female ratio is close to 4:6. In the context of online shopping consumption, the proportion of females is higher than that of males, and females pay more attention to the pursuit of ritual sense than males. Therefore, the male-to-female ratio in the sample is consistent with reality. In terms of age, the age range of 20–30 has the largest number, accounting for 67.2% of the total subjects, indicating that the post-90s’ and post-00s’ generations have become the main force behind online shopping consumption, who are more enthusiastic about exploring new things, which is in line with the intergenerational distribution of online shopping age. In terms of education, 88.5% of the total subjects have a bachelor’s degree or above, indicating that the subjects generally have a high level of education and are able to understand the experimental materials well and ensure quality when filling in the questionnaire. In terms of occupation, 90.8% of the total subjects are enterprise employees, students, civil servants, and institution staff. From the perspective of monthly average consumption, the distribution of the levels above CNY 1000 is relatively even. Therefore, the sample has a good representativeness.

- Reliability and validity analysis

Reliability analysis is conducted on negative emotions, ritual perception, and online word-of-mouth communication. The results show that the coefficient of Cronbach’s α for negative emotions is 0.837, the coefficient of Cronbach’s α for ritual perception is 0.834, and the coefficient of Cronbach’s α for online word-of-mouth communication is 0.811, all greater than 0.8. The CITC value of each item is greater than 0.5, and the coefficients of Cronbach’s α decrease after deleting each item, indicating that three scales used in this study have good reliability.

Subsequently, exploratory factor analysis is conducted using dimensionality reduction functionality. Firstly, negative emotions, ritual perception, and online word-of-mouth communication are tested separately, with KMO values of 0.809, 0.724, and 0.761, respectively, all greater than 0.7 and the significance of corresponding Bartlett’s Test of Sphericity is 0.000 for all, indicating suitability for factor analysis. Secondly, a principal component analysis of the three variables’ items is conducted, and it is found that the factor loading of each item is greater than 0.6 and there is no cross-loading phenomenon, indicating that all items can be retained. Meanwhile, the cumulative-variance explanation rate by the extracted factors is greater than 50%, indicating that the extracted factors can effectively explain the variables. In addition, the AVE value of each variable is all greater than 0.5, and the CR value is all greater than 0.8, indicating that the convergence validity of the three variables is good.

- Manipulation inspection

An independent-samples t-test is conducted for each item and the overall mean of ritual perception. The results show that the overall ritual perception in the brand ritual group is significantly higher than that in the random action group (M_brand ritual group_ = 5.707, M_random action group_ = 4.575, t = 5.455, *p* < 0.05), and symbolic meaning perception (M_brand ritual group_ = 5.64, M_random action group_ = 4.92, t = 2.972, *p* < 0.05), uniqueness perception (M_brand ritual group_ = 5.64, M_random action group_ = 4.09, t = 6.511, *p* < 0.05) and ritualistic color perception (M_brand ritual group_ = 5.85, M_random action group_ = 4.71, t = 4.468, *p* < 0.05) in the brand ritual group are significantly higher than those in the random action group. The ritual in the formal experiment is successfully manipulated.

- Hypothesis testing

**Figure 2:**
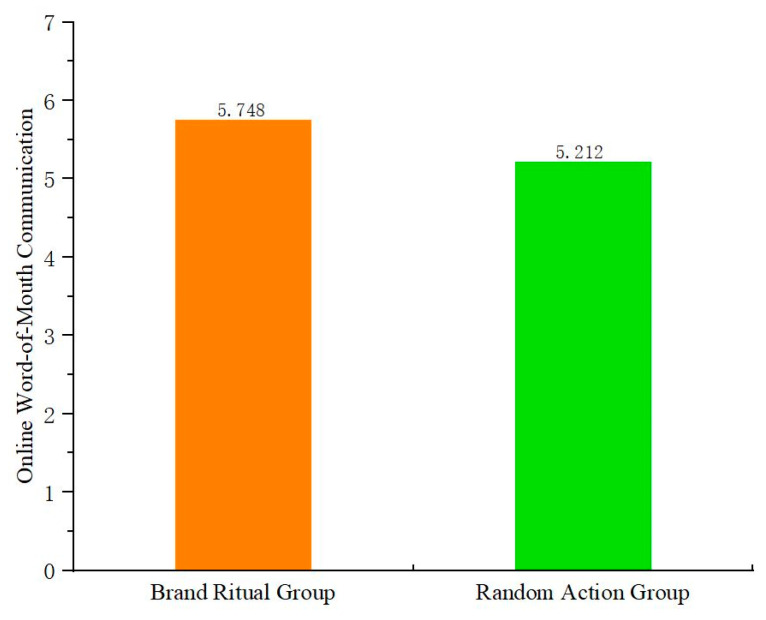
The main effect of brand ritual on online word-of-mouth communication (Experiment 1).

The study tests the main effect of brand ritual on online word-of-mouth communication, and conducts one-way ANOVA with brand ritual (vs. random action) as the independent variable and online word-of-mouth communication as the dependent variable. The results (Figure 2) show that the main effect of brand ritual on online word-of-mouth communication is significant (F(1,129) = 12.94, *p* < 0.05). Compared to random action, brand ritual is more effective in promoting online word-of-mouth communication. Therefore, H1 is verified.

The study eliminates the potential impact of negative emotions, and conducts a general linear model analysis with brand ritual (vs. random action) as the independent variable, online word-of-mouth communication as the dependent variable, and negative emotions as the covariate. The results show that negative emotions have no significant positive impact on the main effect (F(1,128) = 0.893, *p* > 0.1).

### 3.3. Experiment 2

Experiment 2 adopts a single-factor intergroup design (brand ritual vs. random action) to test the mediating role of flow experience in the impact of brand ritual on online word-of-mouth communication, repeatedly verifying the main effect of brand ritual on online word-of-mouth communication in Experiment 1.

#### 3.3.1. Pre-Experiment in Experiment 2

The pre-experiment aims to test the effectiveness of brand ritual manipulation when using the tea bag as the experimental product and the online press conference as the online scene. The experimental group (brand ritual group) and the control group (random action group) use the same brand introduction. The materials are as follows:

**Mingting** is a tea brand with a profound cultural heritage in China, offering multiple types of tea including green tea, black tea, oolong tea, white tea, black tea, and so on. Recently, Mingting has launched a portable tea bag aimed at providing consumers with a more lightweight way to enjoy tea. To better promote the new product, the brand holds an online press conference to promote the new tea bag and introduce its usage.

Subsequently, there is manipulation of the ritual, with both sets of materials strictly limited to 113 words. In addition, the brand ritual group enhances subjects’ ritual perception through the expression of ritualistic behavior, while the random action group improves subjects’ sense of randomness through expressions such as “should” and “should not”. The materials are as follows:

**Brand** **Ritual Group:**

Take out the tea bag, hold the label at the end of the cotton thread and place it at the bottom of the cup.

Slowly pour hot water along the cup wall, lift the tea bag up and down five times, and observe the color change of the tea soup.

Wait for the tea soup to darken in color, fan the air from the cup mouth into your nose, and carefully smell the aroma emitted by the tea soup.

Lift the tea bag and hang it directly above the rim of the cup, carefully observing the ripples caused by the dripping tea soup.

The ritual is completed; let’s start your tea tasting journey.

**Random** **Action Group:**

To ensure the aroma and taste of the tea soup, should use the ceramic cups or glass cups for brewing.

The label at the end of the cotton thread should be placed on the outside of the cup mouth, and should not be brewed together with the tea bag to avoid accidental ingestion.

Inject 200 to 300 milliliters of hot water, and the water temperature should be controlled between 75 °C and 85 °C.

The soaking time should not be too long, and 2 to 3 minutes is the best for taste.

Should not discard the tea bag after use; the tea bag should be placed in the original packaging bag for disposal.

The pre-experiment recruited 73 subjects through a paid recruitment platform (Credamo) and eliminated questionnaires that failed attention detection, obtaining 70 valid questionnaires (29 males and 41 females). The subjects were randomly divided into two groups: 36 in the brand ritual group and 34 in the random action group. After reading the material, the subjects answered relevant questions, including attention detection questions, ritual perception questions, and demographic information questions. The analysis results of the independent-samples *t*-test show that the mean value of ritual perception in the brand ritual group was significantly higher than that in the random action group (M_brand ritual group_ = 5.372, M_random action group_ = 4.571, t = 3.55, *p* < 0.05), the mean value of random perception in the brand ritual group was significantly lower than that in the random action group (M_brand ritual group_ = 2.44, M_random action group_ = 3.26, t = −2.455, *p* < 0.05). The manipulation of brand ritual experimental materials was initially successful.

#### 3.3.2. Formal Experiment in Experiment 2

Firstly, to avoid the potential impact of negative emotions on the subjects, negative emotions are measured before the start of the experiment. Subsequently, the subjects are randomly divided into two groups (brand ritual vs. random action) and read relevant experimental materials separately. Finally, they fill out the questionnaire items based on the materials, including attention detection, ritual perception, flow experience, online word-of-mouth communication, and demographic information. The variables are measured using the 7-point Likert scale. Negative emotions are measured using the scale used in Experiment 1. Ritual perception is measured using the scale used in Experiment 1, with the advertisement replaced by the online press conference. Online word-of-mouth communication is measured using the scale used in Experiment 1, with the watch replaced by the tea bag. The measurement of flow experience is designed based on the research of Zhang et al. (2014), with a total of 5 items (Table 5).

A total of 185 subjects are recruited through a paid recruitment platform (Credamo), the platform’s function of filtering specific questionnaire users is used to ensure that the subjects in the pre-experiment cannot participate in this experiment. Excluding questionnaires that fail attention detection, 181 valid questionnaires are obtained (90 in the brand ritual group and 91 in the random action group). Firstly, the brand ritual group and random action group are coded (0 = random action group, 1 = brand ritual group). Secondly, the negative emotion measurement items, ritual perception measurement items, flow experience measurement items, and online word-of-mouth communication measurement items are averaged. Finally, data analysis is conducted using SPSS 26.0.

- Descriptive statistical analysis

From the perspective of subject gender, the male-to-female ratio is close to 4:6, which is consistent with the male-to-female ratio in online shopping. In terms of age, young people aged 20–30 account for 53.6% of the total subjects, and the sample age distribution is relatively wide, which is basically consistent with the age distribution of online shopping. In terms of education, 89.5% of the total subjects have a bachelor’s degree or above, indicating that the subjects generally have a high level of education and are able to understand the experimental materials well and ensure quality when filling in the questionnaire. In terms of occupation, 96.1% of the total subjects are enterprise employees, students, civil servants, and institution staff. From the perspective of monthly average consumption, there is a higher consumption group above CNY 4000, and each consumption group is involved. Therefore, the sample has a good representativeness.

- Reliability and validity analysis

Reliability analysis is conducted on negative emotions, ritual perception, flow experience, and online word-of-mouth communication. The results show that the coefficient of Cronbach’s α for negative emotions is 0.887, the coefficient of Cronbach’s α for ritual perception is 0.810, the coefficient of Cronbach’s α for flow experience is 0.892, and the coefficient of Cronbach’s α for online word-of-mouth communication is 0.842, all greater than 0.8. The CITC value of each item is greater than 0.5, and the coefficients of Cronbach’s α decrease after deleting each item, indicating that four scales used in this study have good reliability.

Subsequently, exploratory factor analysis is conducted using dimensionality reduction functionality. Firstly, negative emotions, ritual perception, flow experience, and online word-of-mouth communication are tested separately, with KMO values of 0.873, 0.709, 0.868, and 0.793, respectively, all greater than 0.7 and the significance of the corresponding Bartlett’s Test of Sphericity is 0.000 for all, indicating suitability for factor analysis. Secondly, a principal component analysis of the four variables’ items is conducted, and it is found that the factor loading of each item is greater than 0.6 and there is no cross-loading phenomenon, indicating that all items can be retained. Meanwhile, the cumulative-variance explanation rate by the extracted factors is greater than 60%, indicating that the extracted factors can effectively explain the variables. In addition, the AVE value of each variable is all greater than 0.5, and the CR value is all greater than 0.8, indicating that the convergence validity of the four variables is good.

- Manipulation inspection

An independent-samples t-test is conducted for each item and the overall mean of ritual perception. The results show that the overall ritual perception in the brand ritual group is significantly higher than that in the random action group (M_brand ritual group_ = 5.611, M_random action group_ = 4.755, t = 4.828, *p* < 0.05), and symbolic meaning perception (M_brand ritual group_ = 5.47, M_random action group_ = 4.96, t = 2.560, *p* < 0.05), uniqueness perception (M_brand ritual group_ = 5.44, M_random action group_ = 4.18, t = 5.467, *p* < 0.05), and ritualistic color perception (M_brand ritual group_ = 5.92, M_random action group_ = 5.13, t = 3.955, *p* < 0.05) in the brand ritual group are significantly higher than those in the random action group. The ritual in the formal experiment is successfully manipulated.

- Hypothesis testing

(1) Testing the main effect of brand ritual on online word-of-mouth communication

**Figure 3:**
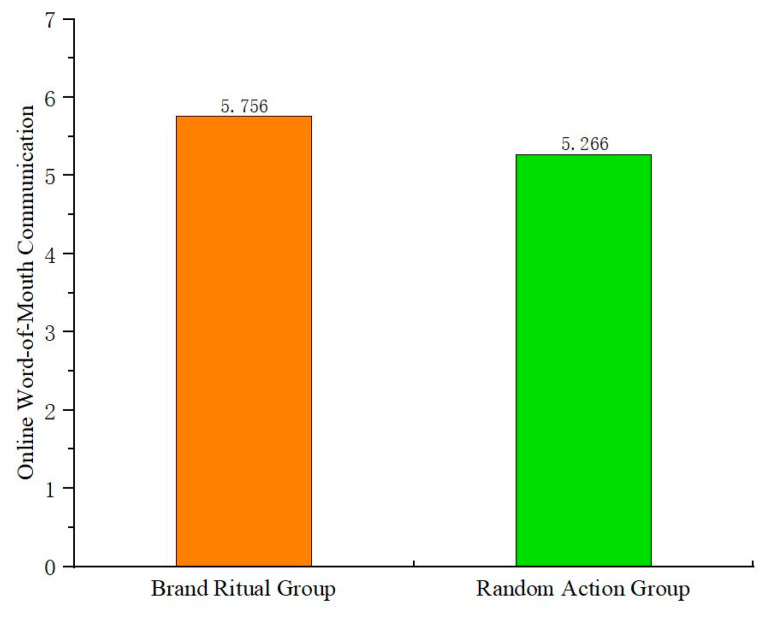
The main effect of brand ritual on online word-of-mouth communication (Experiment 2).

The study conducts one-way ANOVA with brand ritual (vs. random action) as the independent variable and online word-of-mouth communication as the dependent variable. The results (Figure 3) show that the main effect of brand ritual on online word-of-mouth communication is significant (F(1,179) = 11.789, *p* < 0.05). Therefore, H1 is verified again. Using negative emotions as covariates for general linear model analysis, it is found that their impact on the main effect is not significant (F(1,178) = 1.31, *p* > 0.1). The potential effects of negative emotions are ruled out again.

(2) Testing the mediating role of flow experience on the impact of brand ritual on online word-of-mouth communication

**Figure 4:**
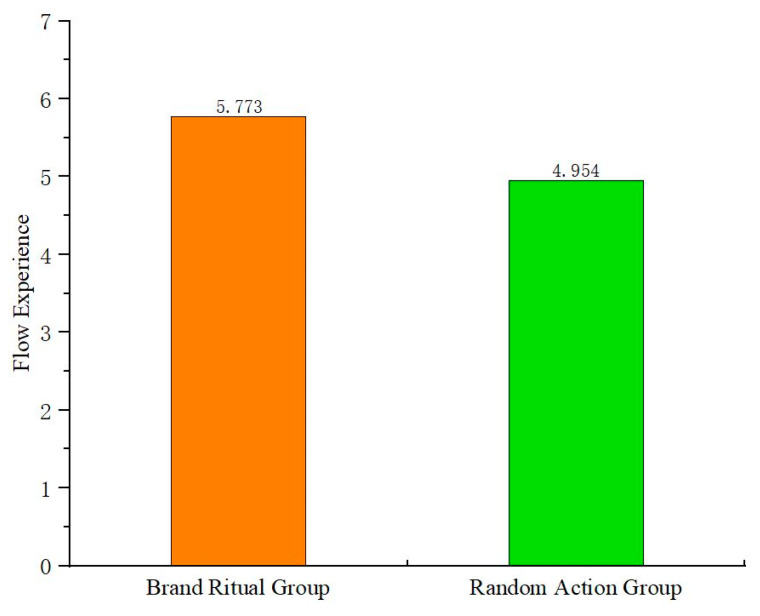
The impact of brand ritual on flow experience (Experiment 2).

Firstly, one-way ANOVA is conducted with brand ritual (vs. random action) as the independent variable and flow experience as the dependent variable. The results (Figure 4) show that brand ritual has a significant positive impact on flow experience (F(1,179) = 27.851, *p* < 0.05). Compared to random action, brand ritual is more effective in stimulating flow experience; H2a is verified. The study conducts one-way ANOVA with flow experience as the independent variable and online word-of-mouth communication as the dependent variable. The results show that flow experience has a significant positive impact on online word-of-mouth communication (F(25,155) = 12.129, *p* < 0.05), and H2b is verified.

Secondly, the study uses the Bootstrap Method of Hayes (2017) to test the mediating role of flow experience. Using the Process (Version 3.5) plugin of SPSS 26.0, the study selects Model 4 and randomly samples 5000 times with the 95% confidence interval. Bootstrap analysis is conducted with online word-of-mouth communication as the dependent variable, brand ritual (vs. random action) as the independent variable, and flow experience as the mediating variable. The result indicates that the indirect effect is 0.4418, with a confidence interval of [0.2277, 0.7033], which does not include 0; after controlling for the mediating variable (flow experience), the direct effect of brand ritual on online word-of-mouth communication is not significant, with the confidence interval of [−0.1978, 0.2934], which includes 0. The above indicates that flow experience plays a completely mediating role, and H2 is verified.

Finally, the study eliminates the potential impact of negative emotions. Using negative emotions as covariates for general linear model analysis, it is found that they are not significant in the effect of brand ritual on flow experience (F(1,178) = 0.912, *p* > 0.1) and flow experience on online word-of-mouth communication (F(1,154) = 0.092, *p* > 0.1). The study adds negative emotions as covariates to conduct Bootstrap analysis. The results indicate that flow experience still plays a completely mediating role. Therefore, the potential effects of negative emotions are ruled out.

### 3.4. Experiment 3

Experiment 3 adopts the two-factor intergroup design of 2 (ritual manipulation: brand ritual vs. random action) × 2 (consumer–brand relationship norms: communal type vs. exchange type) to test the moderating role of consumer–brand relationship norms in the impact of brand ritual on online word-of-mouth communication/flow experience, repeatedly verifying the main effect of brand ritual on online word-of-mouth communication in Experiment 1, and the mediating role of flow experience in the impact of brand ritual on online word-of-mouth communication in Experiment 2.

#### 3.4.1. Pre-Experiment in Experiment 3

The pre-experiment aims to test the effectiveness of brand ritual manipulation when using the scented candle as the experimental product and the promotional video of the e-commerce store as the online scene. The experimental group (brand ritual group) and the control group (random action group) use the same brand introduction. The materials are as follows:

**Xiangyun** is a well-known brand deeply rooted in the field of fragrance, which includes three series of perfume, skincare, and home products. The brand’s philosophy is to blend fragrance with art and share time with you. Through different product designs and atmosphere creation, it brings consumers moments of quiet enjoyment in life. Recently, the brand has launched a wind-chime-scented candle as the main product of the season, and has produced a promotional video to be played repeatedly in its e-commerce store.

Subsequently, there is manipulation of the ritual, with both sets of materials strictly limited to 72 words. Afterwards, the brand ritual group enhances subjects’ ritual perception through the expression of ritualistic behavior, while the random action group improves subjects’ sense of randomness through expressions such as “should”. The materials are as follows:

**Brand Ritual Group:**

Light the candle, put your hands above the flame, and feel the flickering candlelight.

Close your eyes, take three deep breaths, and feel the fragrance emanating.

Fold your hands together and meditate, recalling the experiences of the day.

Use a candle cover to extinguish the candle and hold it for five seconds, then lift it up.

Carefully observe the mist dissipating, trim and straighten the candle wick, and put the candle away.

**Random Action Group:**

For the first buring, keep burning for 2 h until the candle is evenly heated and melted.

Should store it in a cool or dry place at 15 to 20 °C, avoiding direct sunlight.

Before lighting, trim the wick and keep it at 5 to 6 mm, avoiding producing black smoke when the candle burning.

Use an extinguisher to extinguish the candle, which produces smoke and a burnt smell when blown out.

The pre-experiment recruited 81 subjects (26 males and 55 females) through a paid recruitment platform (Credamo). The subjects were randomly divided into two groups: 41 in the brand ritual group and 40 in the random action group. After reading the material, the subjects answer relevant questions, including attention detection questions, ritual perception questions, and demographic information questions. The analysis results of independent-samples t-test show that the mean value of ritual perception in the brand ritual group was significantly higher than that in the random action group (M_brand ritual group_ = 5.772, M_random action group_ = 4.483, t = 3.598, *p* < 0.05), and the mean value of random perception in the brand ritual group was significantly lower than that in the random action group (M_brand ritual group_ = 2, M_random action group_ = 3.18, t = −2.891, *p* < 0.05). The manipulation of brand ritual experimental materials was initially successful.

#### 3.4.2. Formal Experiment in Experiment 3

Firstly, to avoid the potential impact of negative emotions on the subjects, negative emotions are measured before the start of the experiment. Subsequently, the subjects are randomly divided into two groups (communal type vs. exchange type), and read relevant experimental materials in order to manipulate consumer–brand relationship norms. According to the manipulation method of Aggarwal (2004), the number of words in both sets of materials is strictly controlled at 83 words. Communal relationship norms enhance communal perception of the subjects through expressions such as humanization, warmth, and friendship, while exchange relationship norms enhance exchange perception of the subjects through expressions such as high cost-effectiveness, value for money, and commercial exchange. The materials are as follows:

**Communal Relationship Norms (Communal Type):**

Xiangyun is a well-known fragrance brand which is characterized by high humanization. Whenever there is a special occasion or a moment to relax, you always choose Xiangyun to create a warm atmosphere. You can gain a warm interactive experience by consulting its official website, where they will carefully ask about your preferences and recommend categories that are suitable for you. In addition, you can frequently participate in brand events and subscribe to its magazines. Overall, your relationship with Xiangyun is like a friendship.

**Exchange Relationship Norms (Exchange Type):**

Xiangyun is a well-known fragrance brand which is characterized by high cost-effectiveness. You have purchased a scented candle with a lavender odor, and you are satisfied with the odor and duration of burning, making you feel that it has value for money. You can obtain answers from the service department by consulting its official website. In addition, the brand can promote through promotional activities and advertisements to encourage you to purchase new products. Overall, your relationship with Xiangyun is like a commercial exchange.

After reading, the subjects are required to immerse themselves in the scene described in the material and answer questions about the manipulation test of consumer–brand relationship norms based on their true thoughts. There are 4 items each for communal relationship norms and exchange relationship norms. Subsequently, the subjects are further divided into two groups (brand ritual vs. random action) based on the above grouping, and read materials related to ritual manipulation. Finally, the subjects fill out the remaining questionnaire items. Experiment 3 still adopts the 7-point Likert scale. The measurement of negative emotions, attention detection, ritual perception, flow experience, online word-of-mouth communication, and demographic information refers to Experiment 1 and 2. The measurement of (consumer–brand) relationship norms is designed based on the research of Aggarwal (2004) and Song (2022), with a total of 8 items (Table 6).

A total of 269 subjects are recruited through a paid recruitment platform (Credamo), and the platform’s function of filtering specific questionnaire users is used to ensure that the subjects in the pre-experiment cannot participate in this experiment. Excluding questionnaires that fail attention detection, 260 valid questionnaires are obtained (65 each for the communal-ritual group, communal-random group, exchange-ritual group, and exchange-random group). Firstly, communal relationship norms and exchange relationship norms are coded (0 = exchange type, 1 = communal type), and then the brand ritual group and random action group are coded (0 = random action group, 1 = brand ritual group). Next, the study reverses the values of the items of exchange relationship norms and merges them with the values of the items of communal relationship norms to obtain their mean values. Then, the study averages the negative emotion measurement items, ritual perception measurement items, flow experience measurement items, and online word-of-mouth communication measurement items. Finally, data analysis is conducted using SPSS 26.0.

- Descriptive statistical analysis

From the perspective of subject gender, the male-to-female ratio is close to 4:6, which is consistent with the male-to-female ratio in online shopping. In terms of age, people aged 20–30 and 30–40 account for 84.2% of the total subjects, and the sample age distribution is relatively wide, which is consistent with the age distribution of online shopping. In terms of education, 89.2% of the total subjects have a bachelor’s degree or above, indicating that the subjects generally have a high level of education and are able to understand the experimental materials well and ensure quality when filling in the questionnaire. In terms of occupation, 85.4% of the total subjects are enterprise employees and students. From the perspective of monthly average consumption, there is a higher consumption group above CNY 4000, accounting for 45% of the total subjects, and all consumption groups are involved. Therefore, the sample has a good representativeness.

- Reliability and validity analysis

Reliability analysis is conducted on negative emotions, relationship norms (communal and exchange relationship norms), ritual perception, flow experience, and online word-of-mouth communication. The results show that the coefficient of Cronbach’s α for negative emotions is 0.863, the coefficient of Cronbach’s α for communal relationship norms is 0.938, the coefficient of Cronbach’s α for exchange relationship norms is 0.915, the coefficient of Cronbach’s α for ritual perception is 0.879, the coefficient of Cronbach’s α for flow experience is 0.912, and the coefficient of Cronbach’s α for online word-of-mouth communication is 0.868, all greater than 0.8. The CITC value for each item is greater than 0.6, and the coefficients of Cronbach’s α decrease after deleting each item, indicating that the scales used in this study have good reliability.

Subsequently, exploratory factor analysis is conducted using dimensionality reduction functionality. Firstly, negative emotions, communal and exchange relationship norms, ritual perception, flow experience, and online word-of-mouth communication are tested separately, with KMO values of 0.807, 0.864, 0.840, 0.746, 0.884, and 0.827, respectively, all greater than 0.7 and the significance of corresponding Bartlett’s Test of Sphericity is 0.000 for all, indicating suitability for factor analysis. Secondly, a principal component analysis of the above variables’ items is conducted, and it is found that the factor loading of each item is greater than 0.7 and there is no cross-loading phenomenon, indicating that all items can be retained. Meanwhile, the cumulative-variance explanation rate by the extracted factors is greater than 60%, indicating that the extracted factors can effectively explain the variables. In addition, the AVE value of each variable is all greater than 0.5, and the CR value is all greater than 0.8, indicating that the convergence validity of the above variables is good.

- Manipulation inspection

Firstly, independent samples t-test is conducted on relationship norms. The results show that the mean value in communal type is significantly higher than that in exchange type (M_communal type_ = 5.150, M_exchange type_ = 2.811, t = 19.385, *p* < 0.05). The manipulation of relationship norms is successful. Secondly, the study conducts an independent-samples t-test on ritual perception. The results show that the mean value of ritual perception in the brand ritual group is significantly higher than that in the random action group (M_brand ritual group_ = 5.738, M_random action group_ = 4.185, t = 9.617, *p* < 0.05). The ritual in the formal experiment is also successfully manipulated.

- Hypothesis testing

(1) Testing the main effect of brand ritual on online word-of-mouth communication

**Figure 5:**
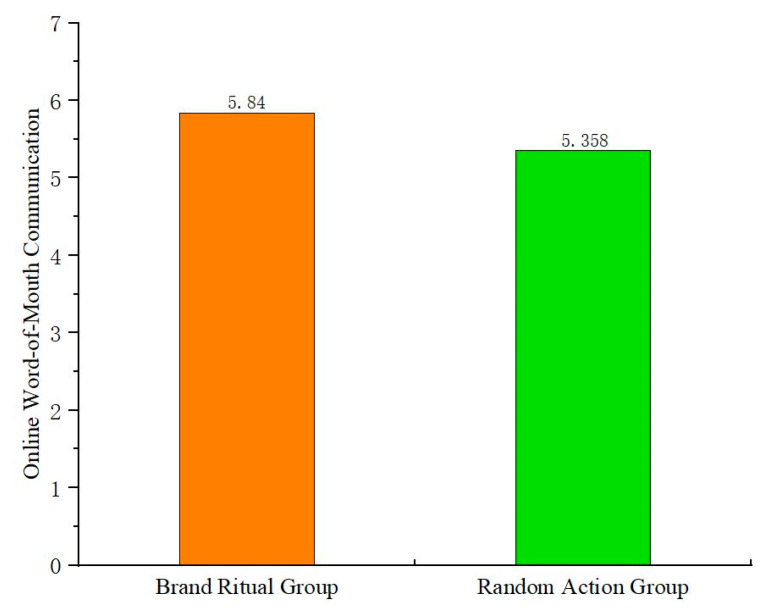
The main effect of brand ritual on online word-of-mouth communication (Experiment 3).

The study conducts one-way ANOVA with brand ritual (vs. random action) as the independent variable and online word-of-mouth communication as the dependent variable. The results (Figure 5) show that the main effect of brand ritual on online word-of-mouth communication is significant (F(1,258) = 16.251, *p* < 0.05). Therefore, H1 is verified again. Using negative emotions as covariates for general linear model analysis, it is found that their impact on the main effect is not significant (F(1,257) = 0.634, *p* > 0.1). The potential effects of negative emotions are ruled out again.

(2) Testing the mediating role of flow experience in the impact of brand ritual on online word-of-mouth communication

**Figure 6:**
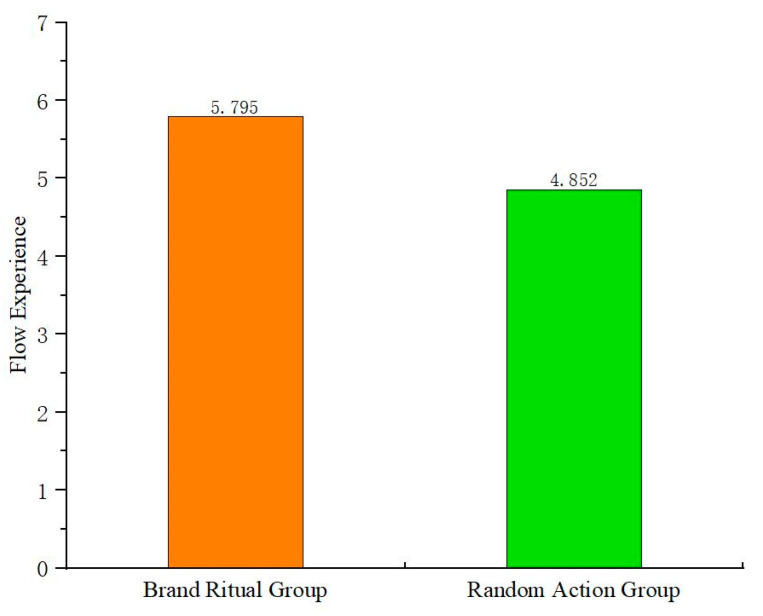
The impact of brand ritual on flow experience (Experiment 3).

Firstly, one-way ANOVA is conducted with brand ritual (vs. random action) as the independent variable and flow experience as the dependent variable. The results show (Figure 6) that brand ritual has a significant positive impact on flow experience (F(1,258) = 45.943, *p* < 0.05). Compared to random action, brand ritual is more effective in stimulating flow experiences; H2a is verified again. The study conducts one-way ANOVA with flow experience as the independent variable and online word-of-mouth communication as the dependent variable. The results show that flow experience has a significant positive impact on online word-of-mouth communication (F(25,234) = 10.243, *p* < 0.05), and H2b is verified again.

Secondly, the Bootstrap Method is used to test the mediating role of flow experience. Using the Process (Version 3.5) plugin of SPSS 26.0, the study selects Model 4 and randomly samples 5000 times with the 95% confidence interval. Bootstrap analysis is conducted with online word-of-mouth communication as the dependent variable, brand ritual (vs. random action) as the independent variable, and flow experience as the mediating variable. The result indicates that the indirect effect is 0.4223, with the confidence interval of [0.2467, 0.6277], which does not include 0; after controlling for the mediating variable (flow experience), the direct effect of brand ritual on online word-of-mouth communication is not significant, with the confidence interval of [−0.1591, 0.2774], which includes 0. The above indicates that flow experience plays a completely mediating role, and H2 is verified again.

Finally, this study eliminates the potential impact of negative emotions. Using negative emotions as covariates for general linear model analysis, it is found that they are not significant in the effect of brand ritual on flow experience (F(1,257) = 0.308, *p* > 0.1) and flow experience on online word-of-mouth communication (F(1,233) = 0.072, *p* > 0.1). The study adds negative emotions as covariates to conduct Bootstrap analysis. The results indicate that flow experience still plays a completely mediating role. Therefore, the potential effects of negative emotions are ruled out.

(3) Testing the moderating role of relationship norms in the impact of brand ritual on online word-of-mouth communication/flow experience

First, this study tests the moderating role of relationship norms in the impact of brand ritual on online word-of-mouth communication. Two-way ANOVA is conducted with brand ritual (vs. random action) and relationship norms (communal type vs. exchange type) as independent variables, and online word-of-mouth communication as the dependent variable. The results show that the interaction between brand ritual and relationship norms is significant (F(1,256) = 39.175, *p* < 0.05), indicating that relationship norms play a moderating role in the impact of brand ritual on online word-of-mouth communication.

**Figure 7:**
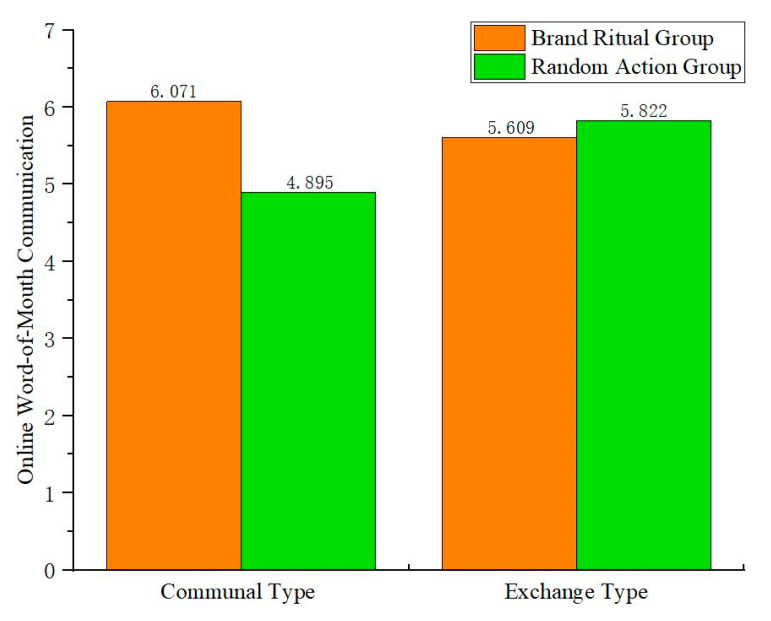
The interactive role of brand ritual and relationship norms on online word-of-mouth communication.

To confirm the stable existence of the interaction, a simple effect test is further used. The results (Figure 7) show that under communal type, brand ritual is more effective in promoting online word-of-mouth communication than random action (F(1,256) = 56.210, *p* < 0.05); under exchange type, there is no significant difference in the impact of random action and brand ritual on online word-of-mouth communication (F(1,256) = 1.834, *p* > 0.1). Therefore, H3a is verified.

Second, the study tests the moderated mediating role of flow experience. 

Firstly, two-way ANOVA is conducted with brand ritual (vs. random action) and relationship norms (communal type vs. exchange type) as the independent variables, and flow experience as the dependent variable. The results show that the interactive role between brand ritual and relationship norms is significant (F(1,256) = 26.512, *p* < 0.05), indicating that relationship norms play a moderating role in the impact of brand ritual on flow experience.

**Figure 8:**
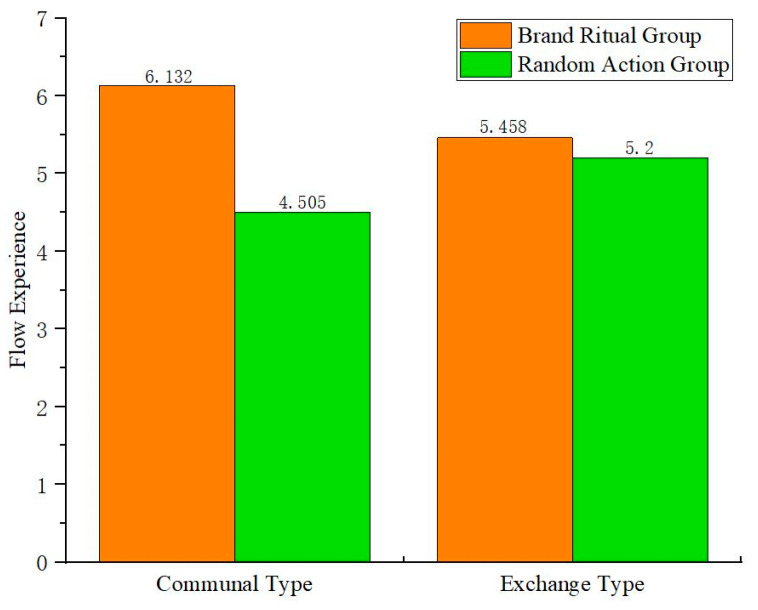
The interactive role of brand ritual and relationship norms on flow experience.

To confirm the stable existence of the interaction, a simple effect test is further used. The results (Figure 8) show that under communal type, brand ritual is more effective in stimulating flow experience than random action (F(1,256) = 74.933, *p* < 0.05); under exchange type, there is no significant difference in the impact of random action and brand ritual on flow experience (F(1,256) = 1.889, *p* > 0.1).

Secondly, using the Process (Version 3.5) plugin of SPSS 26.0, the study selects Model 7 and randomly samples 5000 times, with the 95% confidence interval. Bootstrap analysis is conducted with online word-of-mouth communication as the dependent variable, brand ritual (vs. random action) as the independent variable, flow experience as the mediating variable, and relationship norms (communal type vs. exchange type) as the moderating variable. The results indicate that flow experience plays a significant moderated mediating role, with the confidence interval of [0.2741, 1.0571], which does not include 0. After controlling for the mediating variable (flow experience), the direct effect of brand ritual on online word-of-mouth communication is not significant, with the confidence interval of [−0.1591, 0.2774], which includes 0, indicating that flow experience plays a completely mediating role. Under the communal type, the indirect effect is 0.7289, with the confidence interval of [0.4166, 1.0917], which does not include 0. The indirect effect of flow experience is significant; under the exchange type, the confidence interval is [−0.0662, 0.2696], which includes 0, indicating no significant mediating role.

Overall, under the communal type, brand ritual is more effective in stimulating flow experiences and promoting online word-of-mouth communication than random action; under exchange type, there is no significant difference in the impact of random action and brand ritual on flow experience. Therefore, H3b is verified. Based on the above experimental results, H3a, H3b, and H3 have all been verified.

Finally, the study eliminates the potential impact of negative emotions. Using negative emotions as covariates, the above two-way ANOVA is conducted again, and it is found that their interaction with brand ritual and relationship norms on online word-of-mouth communication (F(1,255) = 1.671, *p* > 0.1) and their interaction on flow experience (F(1,255) = 0.802, *p* > 0.1) are not significant. Subsequently, the study conducts a moderated mediating test. The results show that the role of flow experience is still significant as a moderated mediating variable. The potential effects of negative emotions are ruled out.

## 4. Discussion

### 4.1. Research Findings

#### 4.1.1. Brand Ritual Has a Significant Positive Impact on Online Word-of-Mouth Communication

Three experiments are conducted with the advertisement, online press conference, and promotional video of the e-commerce store as the online scenes, with the watch, the tea bag, and the scented candle as the experimental products, respectively. It is repeatedly found that brand ritual is more effective in promoting online word-of-mouth communication than random action. This research finding once again validates the effectiveness of social exchange theory in the brand marketing field. The existing research has showed that companies can make social-exchange commitments to consumers through the current behavior and situational clues of brand ritual, thereby increasing consumers’ trust and love for the brand (Wei et al., 2020; Cruz-Ros et al., 2024). Different from the existing research, our study focuses on the main effect of brand ritual on online word-of-mouth communication. Undoubtedly, the above finding enriches the relevant research, providing a theoretical basis for companies to use brand ritual to promote online word-of-mouth communication.

#### 4.1.2. Flow Experience Plays a Completely Mediating Role in the Impact of Brand Ritual on Online Word-of-Mouth Communication

Experiments 2 and 3 repeatedly find that flow experience plays a completely mediating role in the impact of brand ritual on online word-of-mouth communication (brand ritual has a significant positive impact on flow experience and flow experience has a significant positive impact on online word-of-mouth communication). In the existing research, Jin and Tian (2016) found that flow experience plays different mediating roles in the impact of website characteristics on online word-of-mouth communication intention, Y. Y. Li and Wang (2021) found that flow experience plays a mediating role in the impact of perceived value on online word-of-mouth communication. However, the two studies did not explore and verify the mediating role of flow experience in the impact of brand ritual on online word-of-mouth communication. From this, it can be seen that the above research finding undoubtedly fills the gap in the existing research.

#### 4.1.3. Consumer–Brand Relationship Norms Play a Moderating Role in the Impact of Brand Ritual on Online Word-of-Mouth Communication or Flow Experience

Experiment 3 finds that consumer–brand relationship norms play a moderating role in the impact of brand ritual on online word-of-mouth communication or flow experience. That is to say, when consumers are guided by communal relationship norms, the impact of brand ritual on online word-of-mouth communication or flow experience is significant. When consumers are guided by exchange relationship norms, the impact of brand ritual on online word-of-mouth communication or flow experience is not significant. The existing research confirmed the moderating role of consumer–brand relationship norms in the impact of apology patterns on post-recovery satisfaction (Lu, 2019), in the impact of social perception evaluation on consumers’ willingness to forgive (Fu, 2021), and in the impact of ritual consumption on self–brand connection (C. Y. Li, 2023). It is undoubtable that the above research findings expand the scope of application of consumer–brand relationship norms, making up for the lack of the existing research.

### 4.2. Managerial Implications

#### 4.2.1. Using Brand Ritual to Promote Consumers’ Online Word-of-Mouth Communication

The ultimate goal of a brand is religion, and rituals are an effective way to fulfill religious beliefs. The study finds that brand ritual can be presented online to consumers through various platforms and methods, and change consumers’ cognition and attitude towards the brand, prompting them to make behaviors that are beneficial to the brand. That is to say, regardless of whether the company is facing participatory consumers or observational consumers, the brand should use the product’s own characteristics as a framework and diverse channels as carriers to construct and spread unique ritualistic activities of the brand, in order to touch, attract, and win over consumers and promote their online word-of-mouth communication.

#### 4.2.2. Stimulating Flow Experience and Promoting Consumers’ Online Word-of-Mouth Communication

With the increasing influence of consumers on online platforms, more and more companies are focusing on shaping and spreading an online reputation. The study finds that brand ritual can stimulate consumers’ flow experience, thereby promoting online word-of-mouth communication. Therefore, when using brand ritual, companies should immerse consumers in a short period of time, provide clear goals and feedback to consumers through a clear ritual process, balance challenges and skills, and encourage exploration and creative thinking, in order to stimulate and promote the formation of highly enjoyable experiences and online word-of-mouth communication.

#### 4.2.3. Grasping Consumer–Brand Relationship Norms and Formulating Brand Marketing Strategies That Are in Line with These Norms

Different types of relationship norms can affect consumers’ expectations when interacting with a brand. Consumers with communal relationship norms (communal type) expect to take part in high-quality emotional interactions and establish a good relationship with the brand; consumers with exchange relationship norms (exchange type) are more inclined to obtain material benefits in the process of interacting with the brand. Therefore, when formulating brand marketing strategies, companies should classify consumers into different types based on their past consumption data. For consumers with communal relationship norms, the strategies promoting brand ritual are effective; for consumers with exchange relationship norms, the strategies providing more material benefits are effective. Based on the above, companies can maximize their brand marketing effect.

### 4.3. Research Limitations and Prospects

Firstly, all three experiments in this study use situational experimental methods to manipulate brand ritual and consumer–brand relationship norms by letting subjects read written materials in order to complete psychological simulations. There may still be some deviation between a single textual expression or psychological simulation method and on-site experiments. Future research will supplement on-site experiments to enhance the external effects of the study.

Secondly, the study only selects three products which can trigger a strong sense of the ritual (the watch, tea bag, and scented candle) for experimental research. Whether the above research findings are applicable to all products requires further verification. Future research will select other products (such as cosmetics, beverages, etc.) to verify the universality of the research findings.

Thirdly, the study only focuses on exploring the positive impact of brand ritual on consumers and brands. However, brand ritual may have negative impacts in certain situations, which is known as the double-edged sword effect (Ran, 2017). Future research will focus on the negative impacts of brand ritual on consumers and brands, and explore the negative effects to help companies avoid marketing risks and build a good interactive ecosystem.

## 5. Conclusions

The study reveals the impact mechanism of brand ritual on online word-of-mouth communication, which is mediated by flow experience and moderated by consumer–brand relationship norms. The study finds that brand ritual has a significant positive impact on online word-of-mouth communication, flow experience plays a completely mediating role in the impact of brand ritual on online word-of-mouth communication, and consumer–brand relationship norms play a moderating role in the impact of brand ritual on online word-of-mouth communication/flow experience. On the basis of the above research findings, companies should learn to use brand ritual to promote consumers’ online word-of-mouth communication, stimulate flow experience and promote consumers’ online word-of-mouth communication, and grasp consumer–brand relationship norms and formulate brand marketing strategies that are in line with these norms.

## Figures and Tables

**Table 1:** Selection of experimental products.

Product Category	Frequency	Gender	
		Male	Female
Tea	21	10	11
Scented candle	18	8	10
Watch	16	9	7
Coffee	14	5	9
Cosmetics	12	1	11
Red wine	11	8	3
Hot pot	10	4	6
Record player	10	6	4

**Table 2:** Measurement scale for negative emotions.

Variable	Number	Measurement Item	Source
Negative emotions (NE)	NE1	Disgust	Xie (2021) Watson et al. (1988)
	NE2	Guilt	
	NE3	Fear	
	NE4	Anger	
	NE5	Nervousness	

**Table 3:** Measurement scale for ritual perception.

Variable	Number	Measurement Item	Source
Ritual perception (RP)	RP1	The series of actions introduced in the advertisement have symbolic significance	Ran (2017)
	RP2	The series of actions introduced in the advertisement feels unique	
	RP3	The series of actions introduced in the advertisement have a ritualistic color	

**Table 4:** Measurement scale for online word-of-mouth communication.

Variable	Number	Measurement Item	Source
Online word-of-mouth communication (OW)	OW1	If my friend wants to buy a watch, I will share and recommend the store or product to him/her through online platforms	Huang (2016) Wang (2021)
	OW2	If my friend is choosing which brand of watch to buy, I will recommend the brand to him/her through online platforms	
	OW3	If my friend is interested, I will share the brand or product information I know with him/her	
	OW4	I will discuss the product information I know in the discussion or comment section of the online platform	
	OW5	I will write down my real experience and comments after purchasing the product	

**Table 5:** Measurement scale for flow experience.

Variable	Number	Measurement Item	Source
Flow experience (FE)	FE1	During the process of interacting with the brand, my curiosity is stimulated	Zhang et al. (2014)
	FE2	During the process of interacting with the brand, my imagination is aroused	
	FE3	The process of interacting with the brand is interesting	
	FE4	The process of interacting with the brand is fun	
	FE5	I am absorbed in the process of interacting with the brand	

**Table 6:** Measurement scale for relationship norms.

Variable	Type	Number	Measurement Item	Source
Relationship norms (RN)	Communal type	RN1	Xiangyun makes me feel warm	Aggarwal (2004) Song (2022)
		RN2	Xiangyun cares about me	
		RN3	I am concerned about the development of Xiangyun	
		RN4	Xiangyun and I are like friends	
	Exchange type	RN5	Xiangyun’s products are worth the money	
		RN6	The purpose of Xiangyun is to obtain business	
		RN7	Xiangyun provides services due to transactions	
		RN8	I have a business relationship with Xiangyun	

## Data Availability

The original contributions presented in the study are included in the article. Further inquiries can be directed to the corresponding author.
